# *GbFLSa* overexpression negatively regulates proanthocyanin biosynthesis

**DOI:** 10.3389/fpls.2023.1093656

**Published:** 2023-02-15

**Authors:** Jing Guo, Yaqiong Wu, Tongli Wang, Yue Xin, Guibin Wang, Qi Zhou, Li-An Xu

**Affiliations:** ^1^ Co-Innovation Center for Sustainable Forestry in Southern China, Nanjing Forestry University, Nanjing, China; ^2^ Institute of Botany, Jiangsu Province and Chinese Academy of Sciences, Nanjing, China; ^3^ Department of Forest and Conservation Sciences, Faculty of Forestry, University of British Columbia, Vancouver, BC, Canada; ^4^ Forest Breeding Institute, Zhejiang Academy of Forestry, Hangzhou, China

**Keywords:** flavonol synthase, gene expression, flavonoid biosynthesis, transgenic poplar, metabolite

## Abstract

Flavonoids are important secondary metabolites with extensive pharmacological functions. *Ginkgo biloba* L. (ginkgo) has attracted extensive attention because of its high flavonoid medicinal value. However, little is understood about ginkgo flavonol biosynthesis. Herein, we cloned the full-length gingko *GbFLSa* gene (1314 bp), which encodes a 363 amino acid protein that has a typical 2-oxoglutarate (2OG)-Fe(II) oxygenase region. Recombinant GbFLSa protein with a molecular mass of 41 kDa was expressed in *Escherichia coli* BL21(DE3). The protein was localized to the cytoplasm. Moreover, proanthocyanins, including catechin, epicatechin, epigallocatechin and gallocatechin, were significantly less abundant in transgenic poplar than in nontransgenic (CK) plants. In addition, *dihydroflavonol 4-reductase*, *anthocyanidin synthase* and *leucoanthocyanidin reductase* expression levels were significantly lower than those of their CK counterparts. GbFLSa thus encodes a functional protein that might negatively regulate proanthocyanin biosynthesis. This study helps elucidate the role of GbFLSa in plant metabolism and the potential molecular mechanism of flavonoid biosynthesis.

## 1 Introduction

Flavonoids are widely distributed in the plant kingdom (Agati et al., 2012). They can be divided into different subgroups depending on the degree of carbon (C) epoxidation and saturation. Flavonoids are important representative secondary products (Agati et al., 2012). A wide variety of flavonoids are composed of 15 carbon atoms arranged in a C_6_-C_3_-C_6_ configuration, i.e., two aromatic rings linked to a C_3_ ring (Satterfield et al., 2001). The biosynthesis of plant flavonoids is a complex process involving a variety of important enzymes. Their synthesis uses p-coumaroyl-CoA and malonyl-CoA as precursors, forming naringenin chalcone under the action of chalcone synthase (CHS), which is the first rate-limiting step in the flavonoid synthesis pathway. The stereospecific cyclization of naringenin chalcone to naringenin is catalyzed by chalcone isomerase (CHI). The conversion of naringin is catalyzed by flavanone 3-hydroxylase (F3H) to produce dihydrokaempferol (DHK), and DHK subsequently forms dihydroquercetin (DHQ) and dihydromyricetin (DHM) (Yonekura-Sakakibara et al., 2019). The above three dihydroflavones can enter the flavonol synthesis pathway under the action of flavonol synthase (FLS), producing kaempferol, quercetin and myricetin. They can also enter the anthocyanin synthesis pathway, which is catalyzed by dihydroflavonol 4-reductase (DFR). Because FLS and DFR catalyze reactions with the same substrate, they exhibit a competitive relationship (Lepiniec et al., 2006). FLS is a key enzyme in the downstream pathway of flavonoid synthesis (Martens et al., 2010). FLS is a dioxygenase that is dependent on iron ions and 2-oxoglutarate (2OG)-dependent dioxygenase (Martens et al., 2010; Cheng et al., 2014). As an intermediate bridge between the flavonoid synthesis pathway and catechin synthesis pathway, FLS is highly conserved in plants. FLSs have been studied in *Petunia hybrida* (Holton et al., 1993), *Nicotiana tabacum* cv Xanthi (Mahajan et al., 2012), *Arabidopsis thaliana* (Pelletier et al., 1997; Owens et al., 2008), and *Oryza sativa* (Park et al., 2019).

*Ginkgo biloba* L. is a perennial deciduous tree that is recognized as a “living fossil” and is one of the oldest relict plants in existing seed plants (Zhou and Zheng, 2003; Gong et al., 2008). Ginkgo is also a typical dioecious gymnosperm with great medicinal, economic and ecological value (Guo et al., 2016; Liu et al., 2016; Wu et al., 2019). The flavonoids in the leaves of ginkgo have a notable effect on the treatment of Alzheimer’s disease, hypertension, and cardiovascular diseases (Ahlemeyer and Krieglstein, 2003; Zhou et al., 2004; Li et al., 2018). Flavonoid contents have become one of the key factors in measuring the quality of Ginkgo biloba extract (GBE) preparations (van Beek and Montoro, 2009; Ye et al., 2019). Determining how to improve flavonoid contents in ginkgo has become a popular research topic (Wu et al., 2018). At present, research on ginkgo flavonoids mainly focuses on their extraction process, medicinal value and pharmacological action (Zhou et al., 2004; Wang et al., 2013; Li et al., 2018). Common methods for improving the yield of ginkgo flavonoids are based mainly on hormone regulation (Xu et al., 2009), optimization of culture conditions (Chen et al., 2014), ultraviolet-B exposure (Zhao et al., 2020), planting density (Lu et al., 2021), and rejuvenation (Lu et al., 2022). Increasing the production of flavonoids by genetic regulation of secondary metabolic pathways is a potential strategy to increase flavonoid production but is difficult due to the lack of complete tissue culture and genetic transformation systems (Dupré et al., 2000; Ayadi and Tremouillaux-Guiller, 2003). The ginkgo genome is very large, and the study of forest tree functional genomics is far less thorough than that of model plants (Guan et al., 2016; Liu et al., 2021). Therefore, further development and utilization of flavonoids to study the functions of key enzymatic genes in the flavonoid biosynthesis pathway is important. Moreover, some new studies have been reported on the molecular mechanisms and specific secondary metabolic pathways in ginkgo flavonoid biosynthesis, such as gene families (Liu et al., 2022), long noncoding RNAs (Liu et al., 2020), and alternative splicing patterns (He et al., 2022). These studies have a certain reference value for understanding the synthesis of flavonoids from other aspects.

FLS is associated with flavonoid accumulation in several plants *in vivo* (Mahajan et al., 2012; Park et al., 2019). However, prokaryotic expression and activity assays of GbFLS have been exclusively performed *in vitro* (Xu et al., 2012), and the specific functions of ginkgo FLS *in vivo* have not been studied. Therefore, based on the ginkgo transcriptome data analysis of flavonoid biosynthesis, transport and regulation (Wu et al., 2018), this study identified 53 candidate enzyme genes related to flavonoid biosynthesis. According to the expression pattern of each enzyme gene and related research, key *F3’5’H* (named *GbF3’5’H1*) (Wu et al., 2020a), *F3’H* (named *GbF3’H1*) (Wu et al., 2020b) and *FLS* (named *GbFLSa*) enzyme genes with relatively high expression levels were finally determined by in-depth research, and this research focused on studying GbFLSa function *in vivo*. This paper reports (i) the isolation and characterization of the *GbFLSa* gene; (ii) differential expression analysis in ginkgo; and (iii) studies of specific functions, including prokaryotic expression, subcellular localization and heterologous overexpression of the *GbFLSa* gene. The aim is to understand the mechanism and metabolic process of flavonoid biosynthesis at the molecular level, which will lay a foundation for further genetic engineering to cultivate plants with related flavonoid compounds.

## 2 Materials and methods

### 2.1 Plant materials

Different tissues, including roots, stems, leaves, kernels, buds, and petioles, were collected from 25-year-old ginkgo trees at Nanjing Forestry University, Jiangsu, China. One-year-old seedlings of ginkgo leaves were obtained from April to October for different expression stages. For functional verification experiments, including transient expression and genetic transformation, we used *Populus davidiana* Dode × *Populus bolleana* Lauche cv. (“Shanxin Yang”) as a control (CK) poplar.

### 2.2 RNA extraction and gene expression analysis

One microgram of total RNA was reverse-transcribed using PrimeScript RT Master Mix (Takara Biomedical Technology Co., Ltd, Beijing, China), and the diluted cDNA (threefold) was used as the template. Then, quantitative real-time fluorescent polymerase chain reaction (RT–qPCR) analysis was carried out using FastStart Universal SYBR Green Master with ROX for RT–PCR Kit (Roche, Indianapolis, IN, USA) to investigate the transcription levels of the *GbFLSa* gene. Finally, all primer sequences and reference genes used for RT–qPCR are listed in **Supplemental Table S1**. Gene expression analysis was performed for ginkgo using the 2^–ΔΔCT^ method (Schmittgen and Livak, 2008) and for *Populus via* the 2^–ΔCT^ method. The glyceraldehyde-3-phosphate and elongation factor 1-α (EF1α) genes were used as reference genes in ginkgo and *Populus*, respectively.

### 2.3 Cloning the GbFLSa gene and bioinformatics analysis

Based on an mRNA fragment annotated in the RNA-seq library of ginkgo (Wu et al., 2018), nested primers were designed to amplify full-length cDNA using the SMARTer RACE 5’/3’ Kit (Clontech, Japan) (**Supplemental Table S1**). Open reading frames (ORFs) were predicted by the NCBI ORFfinder, and the ORF of the *GbFLSa* gene was cloned to further determine its function. Moreover, ExPASy online software, DNAMAN 6.0 software, MEGA 7.0 software, SOPMA software and Gene Structure Display Server online software were used to analyze the FLS protein sequence (Wu et al., 2020a; Wu et al. 2020b).

### 2.4 Prokaryotic expression of GbFLSa in Escherichia coli

The DNA sequence encoding the GbFLSa protein was cloned into pET-30a (+), yielding His-tagged GbFLSa (pET30a-GbFLSa). After sequence confirmation, the resulting recombinant plasmid was introduced into *E. coli* strain BL21 (DE3) *via* the heat shock method (Xu et al., 2012). In brief, sample preparation was as follows: i) Whole cell lysate: Cells were harvested from 200 μL culture and resuspended in 100 μL 5× loading buffer (30% glycerol, 10% SDS, 300 mM Tris, 250 mM DTT). The sample was heated at 100°C for 10 min and centrifuged at 7,000 rpm for 5 min. ii) Supernatant and debris of cell lysate: Cells were harvested from 300 μL culture, lysed with 100 μL BugBuster Protein Extraction Reagent, and incubated at room temperature for 10 min. The cell lysate was centrifuged at 15,000 rpm for 10 min, and the supernatant and cell debris of the cell lysate were collected. Then, 25 μL of 5× loading buffer was added to 100 μL of the cell lysate supernatant. All of the precipitates were resuspended in 50 μL of 5× loading buffer as samples of the debris of the cell lysate. The samples were heated at 100°C for 10 min and centrifuged at 15,000 rpm for 5 min before being loaded into the gel.

### 2.5 Expression vector construction

To obtain a better understanding of the role of the *GbFLSa* gene in flavonoid biosynthesis, we investigated the subcellular localization (Tan et al., 2013) and overexpression of *GbFLSa* in *Populus* (Han et al., 2013). For transient expression assays and overexpression, plasmids were constructed following the methods previously described by Wu et al. (2020b).

### 2.6 Determination and metabolome analysis transgenic *Populus*

After screening using Kan (50 mg/L) resistance, the CK *Populus* and the putative transgenic *Populus* lines were verified by extracting plant DNA using PCR detection and cDNA *via* RT–qPCR. After 30 days of growth, the CK and tested transgenic *Populus* were collected to further study their phenotypes. The plant height and maximum adventitious root length were measured by rulers, and the number of adventitious roots was counted.

Three independent biological replicates from different transgenic lines with higher expression levels in transgenic *Populus* leaves (L2, L5 and L7) and CK plants were used for nontargeted gas chromatography–mass spectrometry (GC–MS) analysis. Each biological replicate consisted of 1-month-old *Populus* leaves from three individual transgenic lines. The determination of metabolites was achieved following the methods previously described by Wu et al. (2020a; 2020b).

To further improve the accuracy of the results of flavonoid-related metabolic substances, we also accurately targeted flavonoids between the CK and transgenic lines through liquid chromatography–mass spectrometry (LC–MS). The details are as follows: 1) Approximately 50 mg of freeze-dried sample was collected, and 600 μL of water: methanol (V: V=1:2) was added, followed by 400 μL of chloroform; the sample was ground with a grinder (60 Hz, 2 min) and then subjected to ultrasonic extraction in an ice water bath for 20 min. 2) The sample was centrifuged for 10 min (4 °C, 13000 rpm), and 500 μL of supernatant was collected into the EP pipe. Next, 400 μL of the residue water: methanol (V: V=1:2) was added, and the sample was vortexed for 1 min and subjected to ultrasonic extraction for 20 min. 3) The sample was centrifuged for 10 min (4 °C, 13000 rpm), and 300 μL of supernatant was collected and consolidated with the previous 500 μL of supernatant for a total of 800 μL. 4) Then, 200 μL of the supernatant was volatilized and added to 200 μL of water:methanol (V:V=18:7) containing 12 ng/mL internal standard (L-2-chlorophenylalanine); the sample was vortexed for 30 s and subjected to ultrasonic extraction for 2 min. 5) The sample was centrifuged for 5 min (4°C, 13000 rpm), and 100 μL of the supernatant was transferred to a brown LC injection bottle and stored at -80°C for future experiments. Quality control samples (QC) were prepared by mixing the extracts of all samples in equal volumes, and the volume of each QC was the same as that of a test sample. UPLC–ESI–MS/MS analysis was used to qualitatively and quantitatively detect the target metabolite. A Waters UPLC HSS T3 (100 × 2.1 mm, 1.7 μm) liquid chromatographic column was used, and the sample injection volume was 5 μL. Mobile phase A was a 0.1% formic acid aqueous solution, and mobile phase B was acetonitrile.

## 3 Results

### 3.1 Characteristics of temporal and spatial expression patterns of GbFLSa

**Figure 1:**
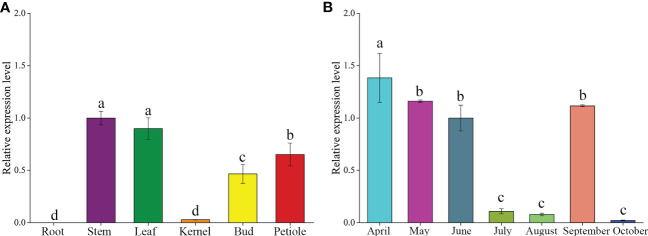
Analysis of temporal and spatial expression levels of the *GbFLSa* gene *via* RT–qPCR. **(A)**. Expression patterns of *GbFLSa* in different ginkgo tissues. **(B)** *GbFLSa* expression in the leaves of ginkgo in different months (from April to October). The y-axis represents the relative expression level, the x-axis of **(A)** represents different tissues, and the x-axis of **(B)** represents different months.

To investigate the potential functions of the *GbFLSa* gene in ginkgo, expression profiles were assessed in different tissues and leaves at different months using RT–qPCR analysis (**Figure 1**). The results showed that *GbFLSa* was expressed in stems, leaves, kernels, buds and petioles but not in roots (**Figure 1A**). The expression level of *GbFLSa* in the stems and leaves was significantly higher than that in other tissues. In addition, expression of *GbFLSa* in the leaves was observed across all months (**Figure 1B**). During the seven months, the expression levels peaked in April and began to decrease from April to August, while October had the lowest expression. Interestingly, the expression in September showed relatively increased levels.

### 3.2 Physicochemical properties, structural characteristics and phylogenetic analysis

To study flavonoid medicinal secondary metabolism and better understand flavonoid biosynthesis, we isolated this key gene using the RACE technique. The full-length cDNA sequence of *GbFLSa* was 1314 bp, including a 13 bp 5’ untranslated region (UTR) and a 209 bp 3’ UTR. This gene contained a 1092 bp ORF with a TAA termination codon encoding a peptide of 363 amino acids. The GbFLSa protein had a predicted molecular weight of 40.93 kDa, a theoretical pI of 6.14, and an instability index of 34.33. This classifies the protein as stable. In addition, the aliphatic index and grand average of hydropathicity were 92.12 and -0.261, respectively.

**Figure 2:**
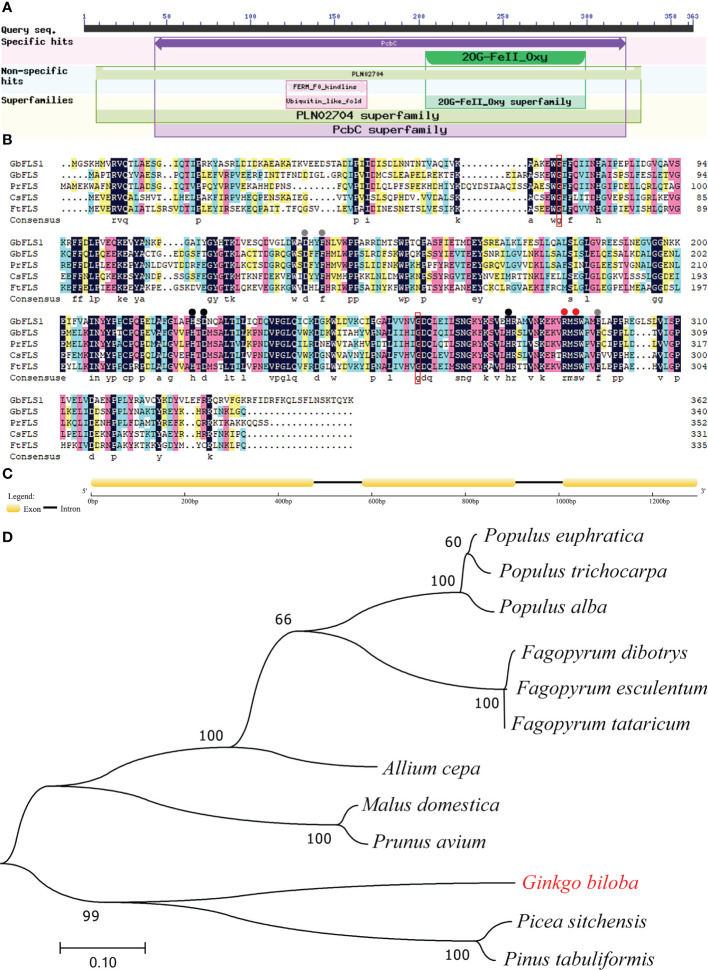
Protein domain, multiple alignment, structural features and phylogenetic analysis of GbFLSa. **(A)**. Protein domain analysis of GbFLSa in ginkgo by NCBI conserved domains. **(B)**. Multiple alignment of the amino acid sequences of GbFLSa and other flavonol synthase (FLS) proteins. GbFLS: *Ginkgo biloba*, ACY00393.1; PrFLS: *Pinus radiata*, AGY80773.1; CsFLS: *Camellia sinensis*, ABM88786.1; and FtFLS: *Fagopyrum tataricum*, AEC33116.1. Red dots show the 2-oxoglutarate-binding residues. The red box indicates the residues responsible for proper folding of the FLS polypeptide. Black dots represent the ferrous iron-binding residues. Gray dots show the putative DHQ-binding residues. **(C)** Exon–intron structure analysis of the *GbFLSa* gene. The exons and introns are represented by boxes and lines, respectively. **(D)** Phylogenetic analysis of GbFLSa and other FLS proteins. The bar indicates a genetic distance of 0.10. GenBank accession numbers are as follows: *Populus euphratica* (XP_011001283.1), *Populus trichocarpa* (XP_002325697.1), *Populus alba* (TKS05742.1), *Fagopyrum dibotrys* (AHN19765.1), *Fagopyrum esculentum* (AEC33115.1), *Fagopyrum tataricum* (AEC33116.1), *Allium cepa* (AQR58516.1), *Malus domestica* (XP_028949987.1), *Prunus avium* (XP_021810338.1), *Picea sitchensis* (ABK26270.1), and *Pinus tabuliformis* (AHW42460.1).

An NCBI conserved domain database search revealed that GbFLSa belongs to the 2OG-Fe(II) oxygenase superfamily (**Figure 2A**). This family contains members of the 2OG- and Fe(II)-dependent dioxygenase superfamily and includes the C-terminus of the prolyl 4-hydroxylase alpha subunit. Comparison of the deduced GbFLSa amino acid sequence and the sequences of other plant FLS proteins in the NCBI database (GbFLS: *Ginkgo biloba*, ACY00393.1; PrFLS: *Pinus radiata*, AGY80773.1; CsFLS: *Camellia sinensis*, ABM88786.1; and FtFLS: *Fagopyrum tataricum*, AEC33116.1) revealed that GbFLSa was conserved (**Figure 2B**). The multiple sequence alignment indicated that all these FLS amino acid sequences have 2OG- (red dots), ferrous iron- (black dots), and putative DHQ-binding residues (gray dots). These sequences also comprise the residues responsible for proper folding of the FLS polypeptide (**Figure 2B** red box). The genomic DNA of the *GbFLSa* gene had two introns and three exons (**Figure 2C**). Moreover, phylogenetic analysis showed that GbFLSa was grouped with gymnosperms close to *Picea sitchensis* (ABK26270.1) and *Pinus tabuliformis* (AHW42460.1) (**Figure 2D**). In addition, all members of the same family of angiosperms were grouped together. In this phylogenetic tree, the relationships between GbFLSa from the analyzed plants generally reflect standard relationships pertaining to gymnosperms and angiosperms.

### 3.3 SDS–PAGE and prokaryotic expression analysis of GbFLSa

**Figure 3:**
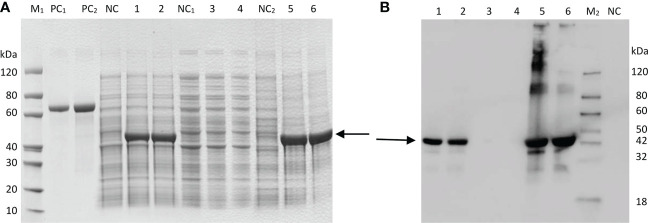
Prokaryotic expression analysis of GbFLSa protein. SDS–PAGE. **(A)** and Western blot **(B)** analysis of GbFLSa cloned in pET-30a (+) and expressed in the BL21 (DE3) strain. Lane M1: protein marker; Lane M2: western blot marker; Lane PC_1_: BSA (1 μg); Lane PC_2_: BSA (2 μg); and Lane NC: cell lysate without induction. Lane 1: cell lysate with induction for 16 h at 15 °C; Lane 2: cell lysate with induction for 4 h at 37 °C; Lane 3: supernatant of cell lysate with induction for 16 h at 15 °C; Lane 4: supernatant of cell lysate with induction for 4 h at 37 °C; Lane 5: debris of cell lysate with induction for 16 h at 15 °C; and Lane 6: debris of cell lysate with induction for 4 h at 37 °C. The arrowheads indicate induced GbFLSa.

SDS–PAGE analysis showed that the target protein could be well expressed *via* the induction of *E. coli* BL21(DE3). After the bacteria were lysed by ultrasonic waves, the supernatant and the precipitate were collected and analyzed by SDS–PAGE. There were obvious specific bands of proteins in the precipitate, while the specific bands in the supernatant were weak, indicating that the recombinant protein was mainly in the form of an inclusion body (**Figure 3A**). The results indicated that the molecular weight of the expressed recombinant protein was 41 kDa, which was consistent with the predicted value (40.93 kDa). The optimal expression conditions were 15°C and induction for 16 h, which provided an experimental basis for further study on the function of GbFLSa. Moreover, western blot analysis of the purified recombinant GbFLSa protein confirmed its specific immune reactivity to anti-His antibodies (**Figure 3B**).

### 3.4 Subcellular localization and heterologous overexpression function analysis

**Figure 4:**
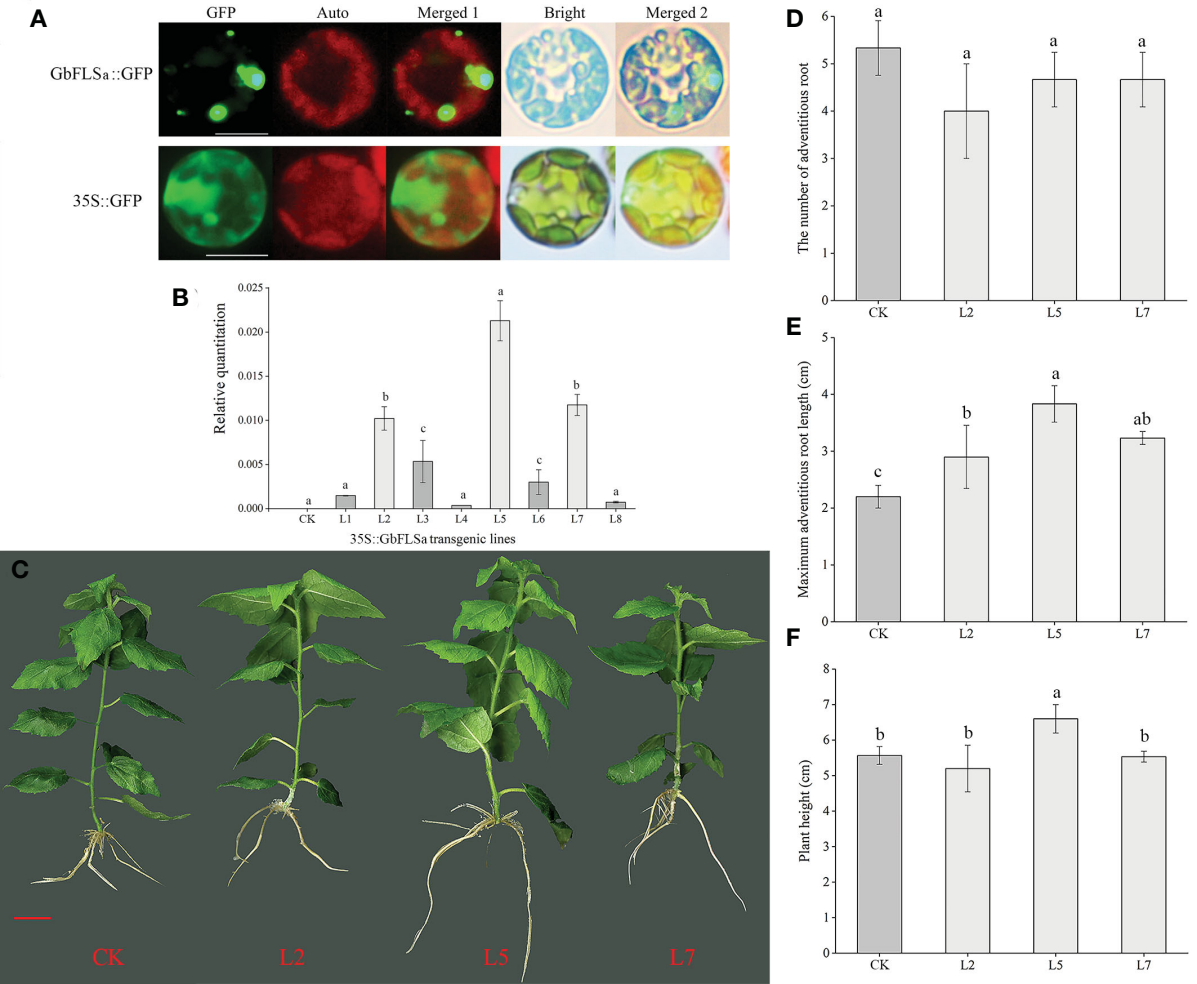
Subcellular localization of the GbFLSa protein and its expression and phenotype comparison analysis of CK *Populus* with transgenic *Populus* lines. **(A)**. Transient expression of the GbFLSa::GFP fusion protein and the 35S::GFP control in *Populus* mesophyll protoplasts. Scale bar, 10 μm. GFP: GFP fluorescence; Auto: chlorophyll autofluorescence; Merged 1: GFP+ Auto; Merged 2: Merged 1+Bright. **(B)**. Relative quantitation analysis of *GbFLSa* transcript levels in CK *Populus* and eight independent transgenic *Populus* lines. **(C)**. Comparison of CK and transgenic *Populus* phenotypes in 30-day-old plants. The number of adventitious roots **(D)**, maximum adventitious root length **(E)** and plant height **(F)** of CK *Populus* and three independent transgenic *Populus* lines. Scale bar = 1 cm. Columns with different lowercase letters significantly (p<0.05) differed based on Duncan’s multiple range test.

To detect the subcellular localization of the *GbFLSa* gene, the control 35S::GFP and GbFLSa::GFP were transformed into *Populus* mesophyll protoplasts. Through confocal microscopy, we found that the GbFLSa::GFP fusion protein was mainly localized in the cytosol (**Figure 4A**); thus, GbFLSa is a cytoplasmic protein. Moreover, to characterize the function of GbFLSa, the full-length ORF of *GbFLSa* was transformed into *Populus* for heterologous expression experiments. Then, to detect whether the *GbFLSa* gene of ginkgo was successfully transferred into the recipient plant genome, PCR amplification was used to detect the DNA level of transgenic plants. The results for 10 transgenic plants were randomly selected and showed that fragments containing target genes were specifically amplified in 8 transgenic poplar lines, indicating that the T-DNA region of the *GbFLSa* recombinant plasmid was successfully integrated into the genomic DNA of *Populus*. No fragments were detected in CK plants. To further detect whether *GbFLSa* was transcribed in transgenic plants, *GbFLSa* expression levels in transgenic *Populus* and CK plants were measured by RT–qPCR. As shown in **Figure 4B**, no *GbFLSa* expression was detected in CK *Populus*, and the expression of *GbFLSa* relative to that of an internal reference gene (*EF1α*) ranged from 0.00036 to 0.02129, among which the L5 line had the highest expression, followed by L7 and L2. Thus, *GbFLSa* was successfully integrated into the recipient plant genome and expressed. In addition, we observed the phenotypes of transgenic and CK plants (**Figure 4C**), including the number of adventitious roots (**Figure 4D**), maximum adventitious root length (**Figure 4E**) and plant height (**Figure 4F**), and some differences in the phenotypes of *GbFLSa* transgenic poplar and CK poplar growth were noted at 30 days of age (**Figure 4C**). Through statistical analysis, the maximum adventitious root length of transgenic *Populus* L2, L5 and L7 was significantly greater than that of CK *Populus* (F=11.931, P=0.003) (**Figure 4E**). Moreover, the transgenic plant L5 was significantly higher than the CK *Populus* plant (**Figure 4F**).

### 3.5 Variations in flavonoid-related metabolites by nontargeted GC–MS and LC–MS metabolic analysis

**Figure 5:**
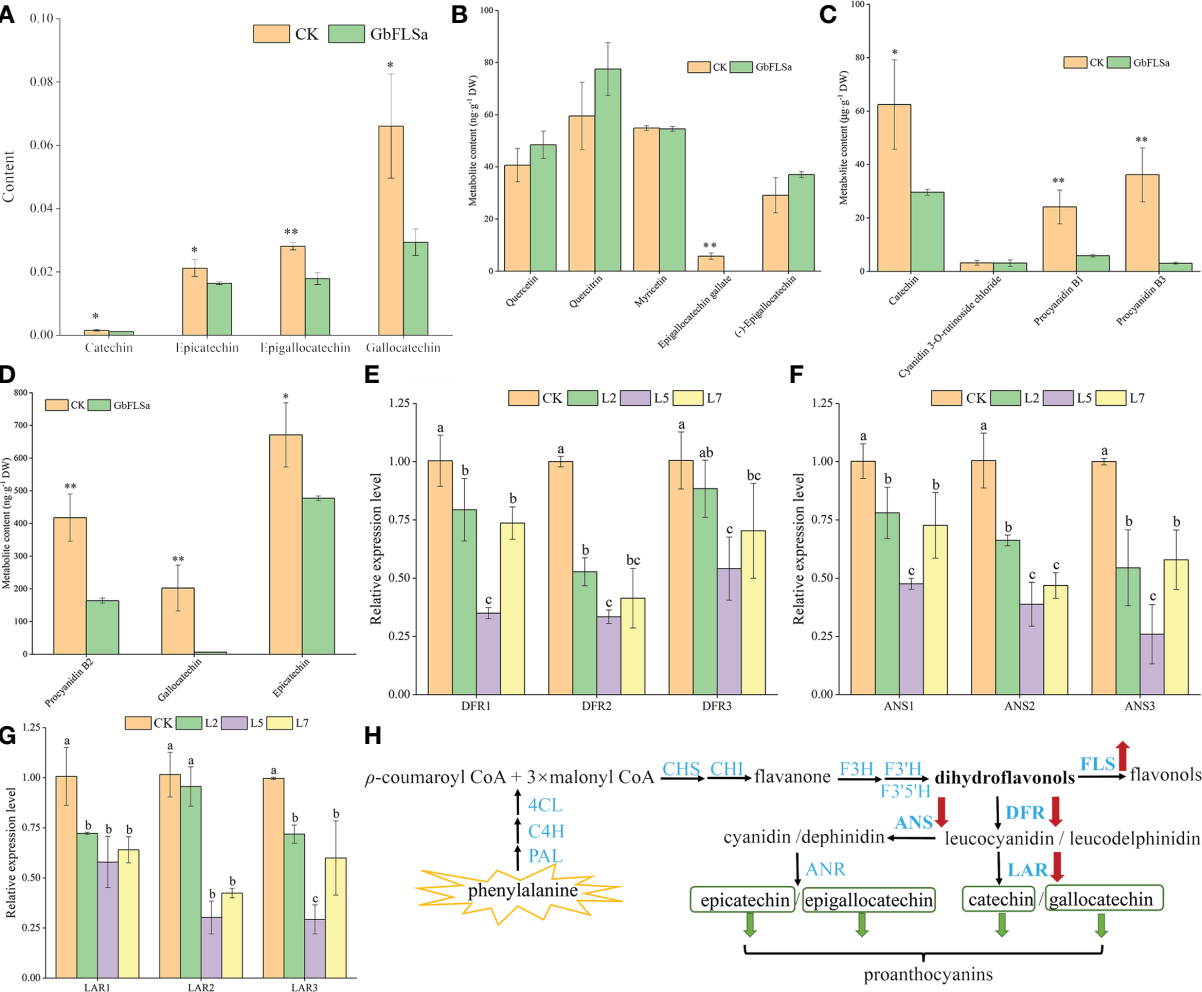
Flavonoid-related metabolite contents, enzyme gene expression levels and their biosynthesis pathways between the CK and transgenic plants. **(A)**. Comparison of four differential metabolite contents *via* GC–MS analysis. Columns with different lowercase letters significantly (p<0.05) differed based on Duncan’s multiple range test. **(B–D)** represent the metabolite content of flavonoids between transgenic and CK plants by LC–MS analysis. **(E–G)** represent the relative expression levels of three enzyme-related genes by RT–qPCR analysis between the nontransgenic *Populus* and transgenic *Populus* groups. **(H)** Flavonoid biosynthesis pathway. * represents P < 0.05; ** represents P < 0.01.

To further determine the differences in metabolites between *GbFLSa* transgenic plants and CK plants, three relatively highly expressing transgenic lines (L2, L5 and L7) and CK poplar were selected for nontargeted GC–MS metabolism detection. The original files were extracted by mass spectrometry, and the peaks of all experimental samples were compared with those in a database. A total of 29 differential metabolites were obtained by the combination of multidimensional and unidimensional analysis, and most of them were primary metabolites (**Supplemental Data 1**). Among them, the levels of 16 differential metabolites (approximately 55%) were decreased in transgenic *Populus* relative to their CK levels. Differential metabolites between CK and transgenic *Populus* included 7 phenylpropanoids and polyketides, among which 4 proanthocyanin metabolites are closely related to the biosynthesis of flavonoids. They are catechin, epicatechin, epigallocatechin and gallocatechin, which belong to the flavan subclass. Metabolite determination showed that the content of these four metabolites in transgenic *Populus* was significantly lower than that in CK *Populus* (**Figure 5A**). The content of gallocatechin in the CK *Populus* was 2.2 times that of transgenic *Populus*. To obtain more accurate results, we also determined flavonoids in transgenic and CK seedlings by LC–MS. The results indicated that the epigallocatechin gallate, catechin, gallocatechin and epicatechin contents in transgenic *Populus* were significantly lower than those in CK *Populus* (**Figures 5B–D**), which was basically consistent with the results of GC–MS analysis. Moreover, we also found that the metabolite contents of procyanidin B1, procyanidin B2 and procyanidin B3 in the CK plants were significantly higher than those in the transgenic *Populus* plants, indicating that GbFLSa overexpression negatively regulates proanthocyanin biosynthesis.

### 3.6 Analysis of DFR, ANS and LAR expression levels in CK and transgenic poplar

To further understand the changes in the expression of genes related to the same substrate shared by FLS in transgenic and CK plants and the expression of genes upstream of related metabolites, we selected three *DFRs*, three *anthocyanidin synthases* (*ANSs*) and three *leucoanthocyanidin reductase* (*LARs*) in the flavonoid biosynthesis pathway for expression detection and analysis. The results showed that the relative expression levels of *DFRs* (*DFR1* and *DFR2*), *ANSs* (*ANS1*, *ANS2* and *ANS3*) and *LARs* (*LAR1* and *LAR2*) in the three transgenic poplar lines were significantly lower than those in the CK poplar (**Figures 5E–G**). Interestingly, the relative expression patterns of *DFRs*, *ANSs* and *LARs* in the CK group were significantly higher than those in the transgenic line L5. Therefore, this result indirectly reflects the decreased content of proanthocyanins (catechin, epicatechin, epigallocatechin and gallocatechin) produced by the enzymes encoded by genes downstream in the flavonoid pathway in transgenic plants (**Figure 5H**).

## 4 Discussion

Given that flavonoids are ubiquitous in plants, complex metabolic pathways play an important role in plant evolution (Weng, 2014). Genomic studies of ginkgo have shown that gene multiplication events occurred in four (*CHS*, *F3H*, *FLS* and *DFR*) enzyme genes in the flavonoid biosynthesis pathway, which partly explained the abundant level of flavonoids in ginkgo (Guan et al., 2016; Liu et al., 2021). Some studies have shown that the composition and content of flavonols are related to plant species, varieties, tissues, developmental stage and growth environment (Koyama et al., 2012; Ye et al., 2019). *FLS* gene expression levels were higher in young leaves than in mature leaves, and the highest level was observed in the mature peel of *Citrus unshiu* Marc. (Moriguchi et al., 2002). *GbFLSa* expression was highest in the stems and leaves in this study, implying that this gene might play important roles in those areas. This result indicated that the expression patterns of *FLS* enzyme genes in different tissues of different plants were significantly different. Lobstein et al. (1991) showed that the flavonoid content in ginkgo leaves was the highest in April. The content of biflavone in ginkgo leaves is the highest in autumn, followed by spring, which may be due to the accumulation of flavonoids before turning yellow in autumn, leading to a peak (Briancon-Scheid et al., 1983). The flavonoid content in ginkgo leaves was higher in spring and autumn. In this study, the expression level of the *GbFLSa* gene was the highest in April, followed by May and September, which is similar to the accumulation pattern of flavonoid content observed in previous studies (Briancon-Scheid et al., 1983; Lobstein et al., 1991). GbFLSa has a typical 2OG-Fe(II) oxygenase activity domain (**Figure 2A**), in which 5 amino-terminal residues in the enzyme activity domain are highly conserved in different species (“H-D-H-R-S”) (**Figure 2B**), which is consistent with sequences reported in other FLS studies (Owens et al., 2008; Toh et al., 2013; Zhou et al., 2013). The genomic DNA of *GbFLSa* has two introns and three exons (**Figure 2C**), which is consistent with Liu et al. (2022)’s study because 90% of GbFLSs have two introns and three exons. The putative splicing site is subject to the GT/AG rules, and the result is the same as that reported by Xu et al. (2012). Previous studies have shown that exons directly encode proteins and that introns influence RNA synthesis during transcription (Mattick and Gagen, 2001). Moreover, the postspliced introns interact with the corresponding coding sequences and play an important role in regulating mRNA transport and gene expression (Le Hir et al., 2003; Zhao et al., 2013; Bo et al., 2019). Therefore, the evolution and functional role of FLS introns remain to be studied in the future. Liu et al. (2022) conducted family analysis on all FLSs in the ginkgo genome and found that there were 10 FLSs in ginkgo, of which seven GbFLSs were distributed in a continuous manner. There are only 6 FLSs in *Arabidopsis*, which shows that the copy number of FLSs in different species is different. Phylogenetic tree results showed that GbFLSa was closely related to FLSs in other gymnosperm species (*P. sitchensis* and *P. tabuliformis*), which was consistent with the classification of plant species (Guan et al., 2016). These evolutionary relationships reflected the diversity of *FLS* genes in plants.

Some studies on flavonoid biosynthesis-related genes in ginkgo are limited to determining enzyme activity *in vitro*, gene expression levels and cooperative analysis of flavonoid content (Xu et al., 2012), and few experiments have confirmed the endogenous functions of these enzyme genes in woody plants. Flavonoids are special secondary metabolites of plants that have antioxidant and antitumor functions and promote blood circulation (Ahlemeyer and Krieglstein, 2003; Zhou et al., 2004; Li et al., 2018). Because ginkgo does not have a complete genetic transformation and regeneration system, this research studied the biological function of the *GbFLSa* enzyme gene in transgenic poplar, a model species of woody plants. The *E. coli* exogenous protein expression system is currently the most commonly used prokaryotic expression system. In this study, expression of a GbFLSa recombinant protein that was consistent with the predicted molecular weight was successfully induced *in vitro*, but this protein exists mainly as an inclusion body, which to some extent limited the research on the catalysis characteristics of the protein. Transient expression analysis of protoplasts is essential for high-throughput screening and systematic characterization of plant gene functions (Xuan et al., 2014). Here, we used a *Populus* protoplast transient expression system to successfully express the GbFLSa protein in the cytoplasm. FLS1 was located in the nucleus of *Arabidopsis thaliana* (Kuhn et al., 2011), which differed from the results of this study. Proteins are the basic unit of life activity, and their structures determine their functions, which indicates that FLSs in different locations perform different functions. At present, *FLSs* have been extensively studied in many plants (Nielsen et al., 2002; Vu et al., 2015; Liu et al., 2022). Liu et al. (2022) analyzed the diversity of cis-acting elements of FLSs in the ginkgo genome and found that light-responsive elements exist in all GbFLS genes, followed by MYC- and MYB-related elements. At the same time, there are some hormone-responsive elements in different GbFLS members, such as auxin-responsive elements, MeJA-responsive elements, SA-responsive elements, GA-responsive elements, and ABA-responsive elements. Relevant studies have shown that phytohormones are key factors in plant tissue culture, which not only affect cell growth but also affect the synthesis of secondary metabolites in plants (Murthy et al., 2014). Therefore, overexpression of the GbFLSa gene in this study may lead to the difference between transgenic and nontransgenic plants in terms of maximum adventitious root length and plant height. FLS is a key enzyme responsible for flavonol synthesis in the flavonoid pathway, which catalyzes the formation of flavonol from dihydroflavonol. The FLS gene plays an important role in regulating flavonoid metabolism in the flavonol and anthocyanin branching pathways (Nielsen et al., 2002). Inhibition of *FLS* gene expression using an antisense RNA technique in *P. hybrida* resulted in enhanced anthocyanin synthesis, increased petal pigmentation (Holton et al., 1993) and a significantly decreased flavonol content (Davies et al., 2003). *FLS* gene expression was inhibited in *Eustoma grandiflorum* Grise, resulting in red flowers, the accumulation of dihydroflavonols, and low levels of flavonol accumulation (Nielsen et al., 2002). After overexpression of the BnFLS gene, flavonol (kaempferol and quercetin) levels were recovered in the Arabidopsis atfls1-ko mutant (Vu et al., 2015). These results indicated that the *FLS* gene was directly related to the synthesis of flavonol. Mahajan et al. (2012) studied the changes in flavonol synthesis content after silencing the *FLS* gene in tobacco. They found that the yield of flavonol was significantly decreased after transgene transfection and that the contents of catechin and epicatechin increased by 50-90% and 20-30%, respectively. All of the above studies indicated that *FLS* plays an important role in regulating flavonoid metabolism, specifically the flavanol and anthocyanin branching pathways. In this study, the content of proanthocyanins (catechin, epicatechin, epigallocatechin and gallocatechin) in transgenic poplar plants was significantly lower than that of the CK group (**Figure 5A**). The expression levels of two *DFRs*, three *ANSs* and two *LARs* were also significantly lower than those in the CK group (**Figures 5E–G**). This indicates that FLS and DFR enzymes compete for the same substrate, and the increased expression of the *FLS* enzyme gene may affect the efficiency of DFR enzyme synthesis, resulting in a decrease in flavonoid (proanthocyanin), which is the downstream product of the DFR enzyme (**Figure 5H**). Luo et al. (2015) also showed that *FLS* and *DFR* were expressed simultaneously, competition existed for the common substrate dihydroflavonols, and FLS activity began to decrease rapidly as proanthocyanins began to be produced. *DFR* expression was positively correlated with proanthocyanin accumulation and negatively correlated with flavonol synthesis, which was consistent with the results of relevant studies (Polashock et al., 2002; Zhou et al., 2013). This finding also indirectly suggests an interaction with flavonoid synthetase, which regulates the structure and type of flavonoids (Fujino et al., 2018). In addition, we also used targeted LC–MS to determine flavonoid-related substances in transgenic poplar and CK poplar and found that the epigallocatechin gallate, catechin, gallocatechin, epicatechin, procyanidin B1, procyanidin B2 and procyanidin B3 contents in transgenic *Populus* were significantly lower than those in CK *Populus* (**Figures 5B–D**), indicating that GbFLSa overexpression negatively regulates proanthocyanin biosynthesis. Some studies have shown that flavonol biosynthesis is regulated by single or multiple transcription factor complexes, such as MYB, bHLH and WD40 (Tohge et al., 2005). The regulatory mechanism by which related transcription factors affect the structural genes of flavonol synthesis remains unknown. In summary, the study of the *GbFLSa* gene provides a basis for elucidating the molecular mechanism and scientific significance of the plant flavonoid metabolic pathway.

## 5 Conclusions

In this study, the full-length *GbFLSa* gene producing a 1314 bp cDNA was successfully cloned from ginkgo and found to encode a 363 amino acid sequence that showed a typical 2OG-Fe(II) oxygenase activity region. The genomic DNA of *GbFLSa* has two introns and three exons. Expression analysis showed that the peak relative expression in leaves occurred in April. Functional studies indicated that the GbFLSa protein is located in the cytoplasm, and the contents of catechin, epicatechin, epigallocatechin and gallocatechin in transgenic poplar were significantly lower than those in the CK group. In addition, the *DFR*, *ANS* and *LAR* expression levels were also significantly lower than their counterparts in the CK group. This result indicates that overexpression of *GbFLSa* plays a negative regulatory role in the biosynthesis of proanthocyanins (catechin, epicatechin, epigallocatechin and gallocatechin).

## Data availability statement

The datasets presented in this study can be found in online repositories. The names of the repository/repositories and accession number(s) can be found in the article/**Supplementary Material**.

## Author contributions

JG and YW: Writing-original draft, Formal analysis; YW and YX: Investigation, Experiment; QZ: Software; GW: Resources; TW and L-AX: Writing-review & editing. All authors have read and agreed to the published version of the manuscript.

## Supplementary material

Supplementary Data Sheet 1 Twenty-nine differentially abundant metabolites between CK and transgenic *Populus.*
