# Supplementary figures and images for: GbFLSa overexpression negatively regulates proanthocyanin biosynthesis
Source: Front Plant Sci. 2023 Feb 15;14:1093656. doi: 10.3389/fpls.2023.1093656 (PMC9975577; doi:10.3389/fpls.2023.1093656)

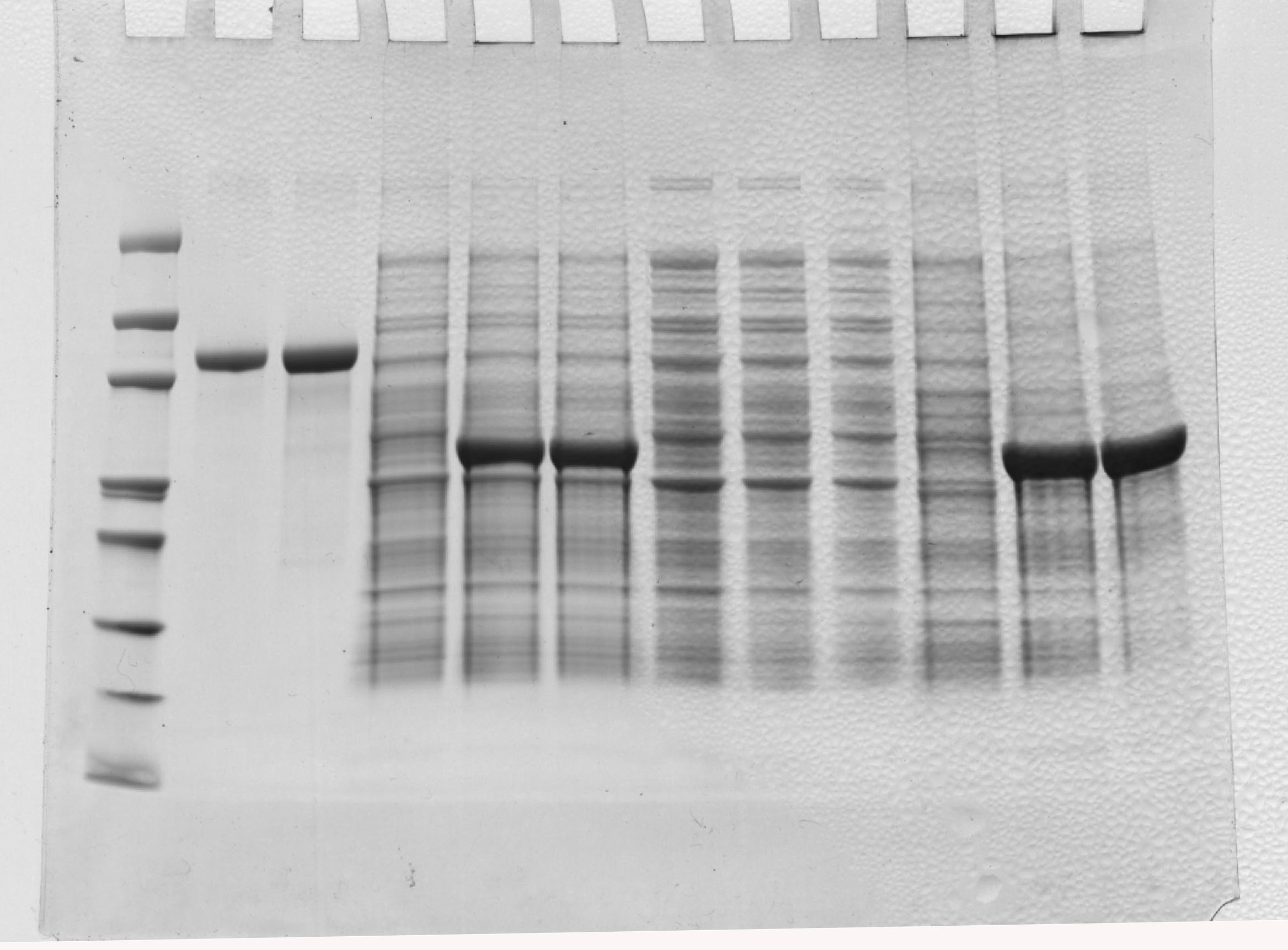

Supplement: Supplementary Data Sheet 1 — Twenty-nine differentially abundant metabolites between CK and transgenic Populus. [file DataSheet_1.zip › Data Sheet 1/Raw Data1093656/Fig.3/SDS.jpg]

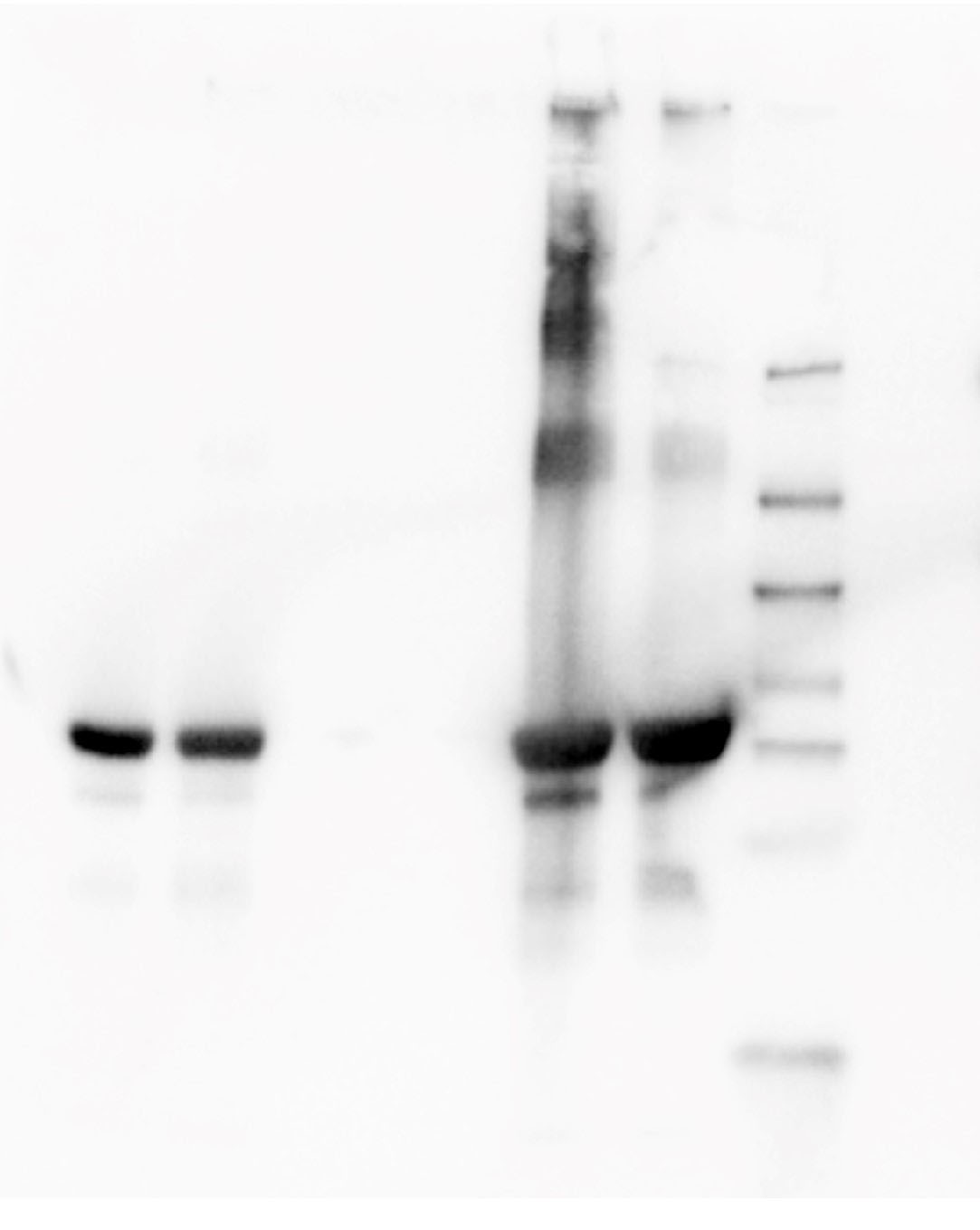

Supplement: Supplementary Data Sheet 1 — Twenty-nine differentially abundant metabolites between CK and transgenic Populus. [file DataSheet_1.zip › Data Sheet 1/Raw Data1093656/Fig.3/WB.jpg]

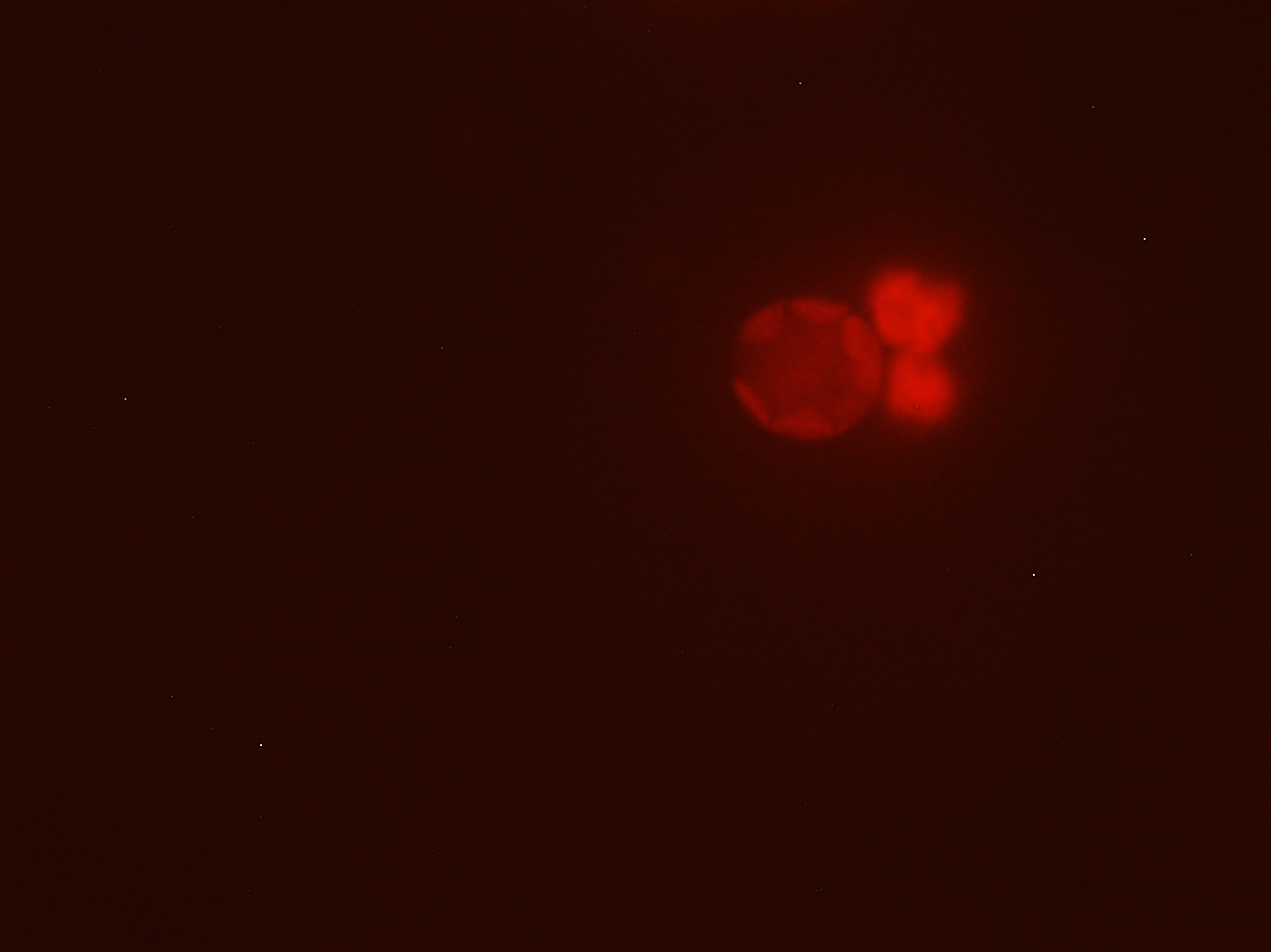

Supplement: Supplementary Data Sheet 1 — Twenty-nine differentially abundant metabolites between CK and transgenic Populus. [file DataSheet_1.zip › Data Sheet 1/Raw Data1093656/Fig.4/35S/┼─╔π1-1018.tif]

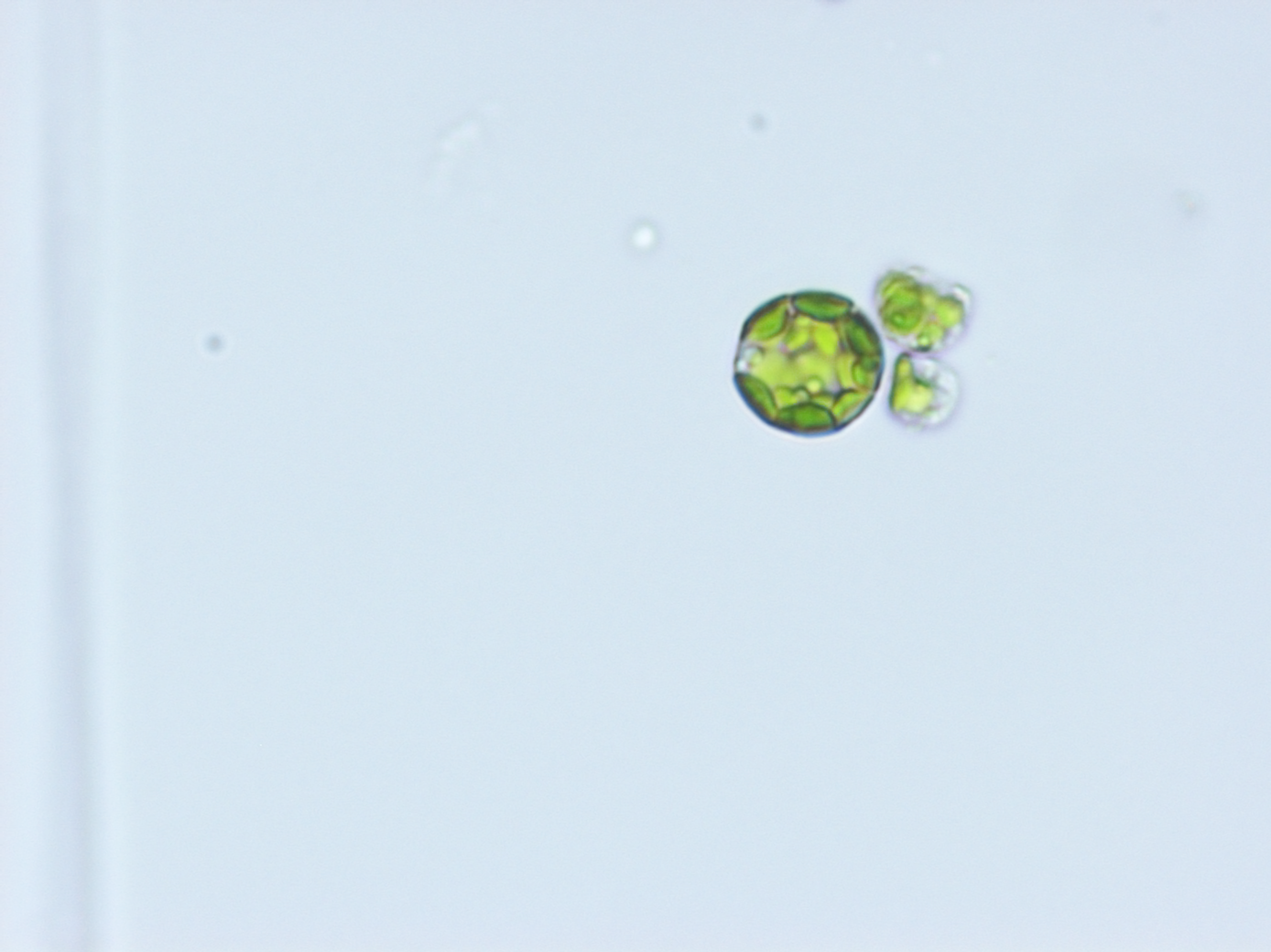

Supplement: Supplementary Data Sheet 1 — Twenty-nine differentially abundant metabolites between CK and transgenic Populus. [file DataSheet_1.zip › Data Sheet 1/Raw Data1093656/Fig.4/35S/┼─╔π1-1019.tif]

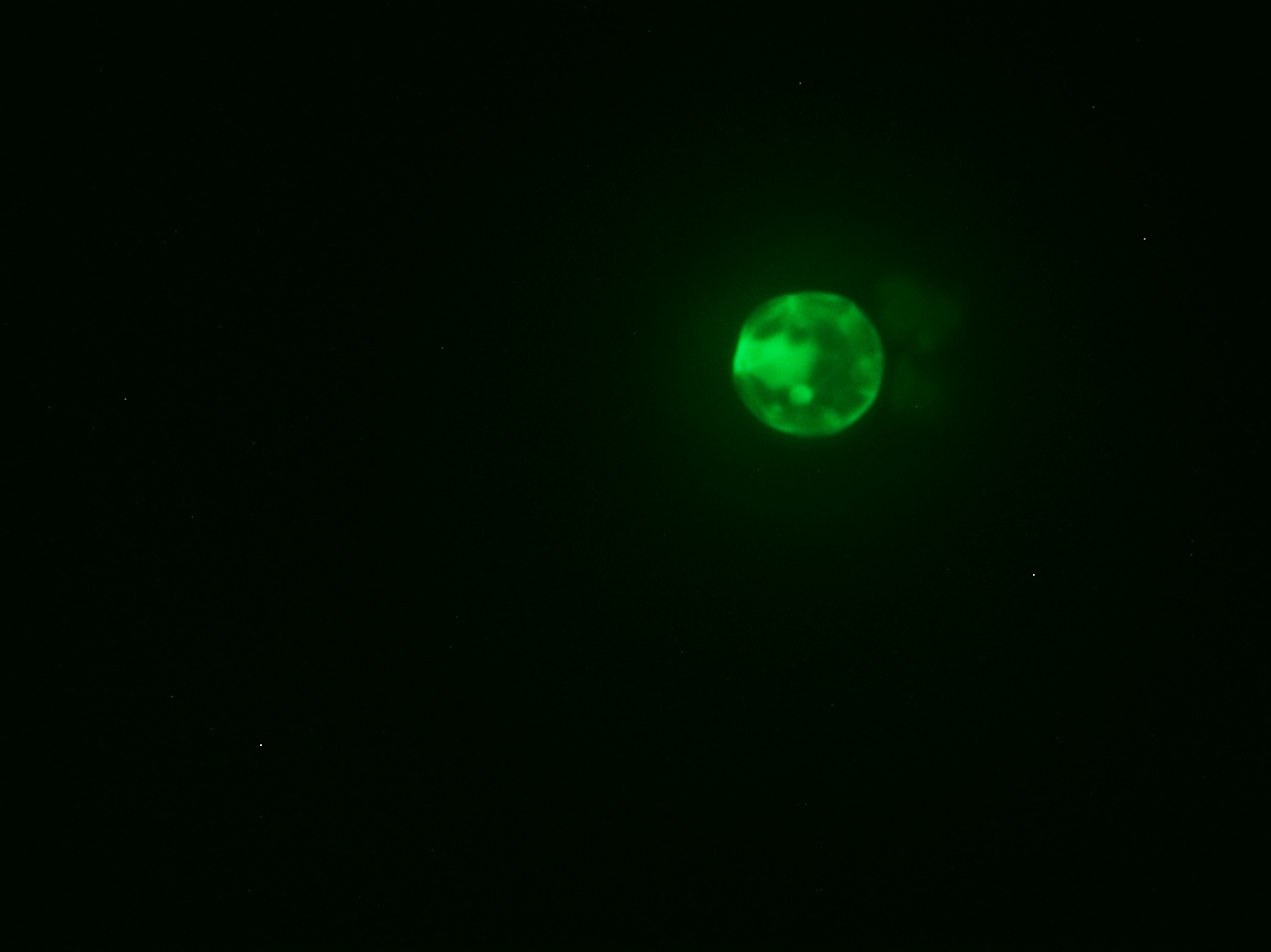

Supplement: Supplementary Data Sheet 1 — Twenty-nine differentially abundant metabolites between CK and transgenic Populus. [file DataSheet_1.zip › Data Sheet 1/Raw Data1093656/Fig.4/35S/┼─╔π1-1020.tif]

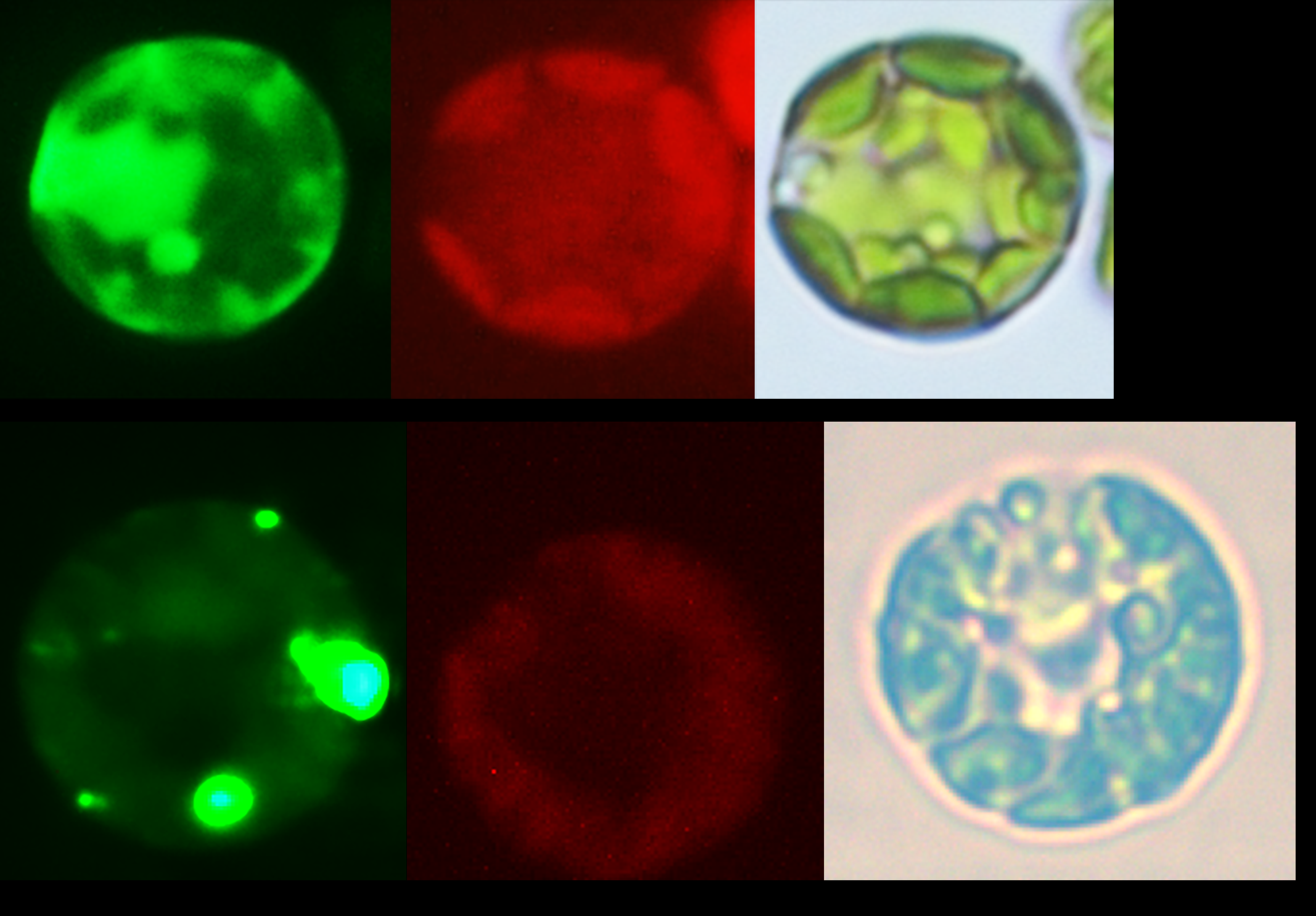

Supplement: Supplementary Data Sheet 1 — Twenty-nine differentially abundant metabolites between CK and transgenic Populus. [file DataSheet_1.zip › Data Sheet 1/Raw Data1093656/Fig.4/35S/┼─╔π1-1020111.tif]

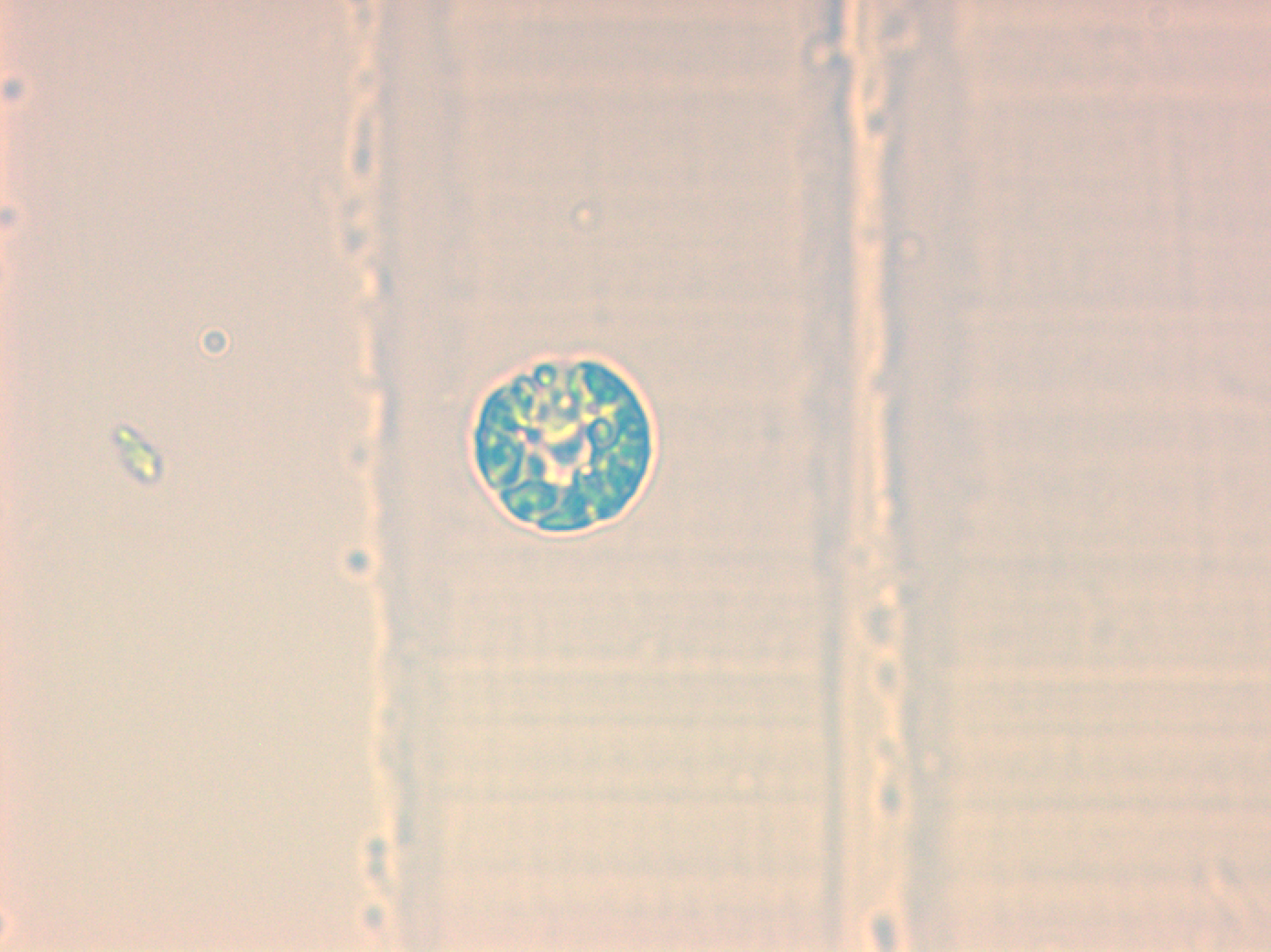

Supplement: Supplementary Data Sheet 1 — Twenty-nine differentially abundant metabolites between CK and transgenic Populus. [file DataSheet_1.zip › Data Sheet 1/Raw Data1093656/Fig.4/FLS/┼─╔π-1631.tif]

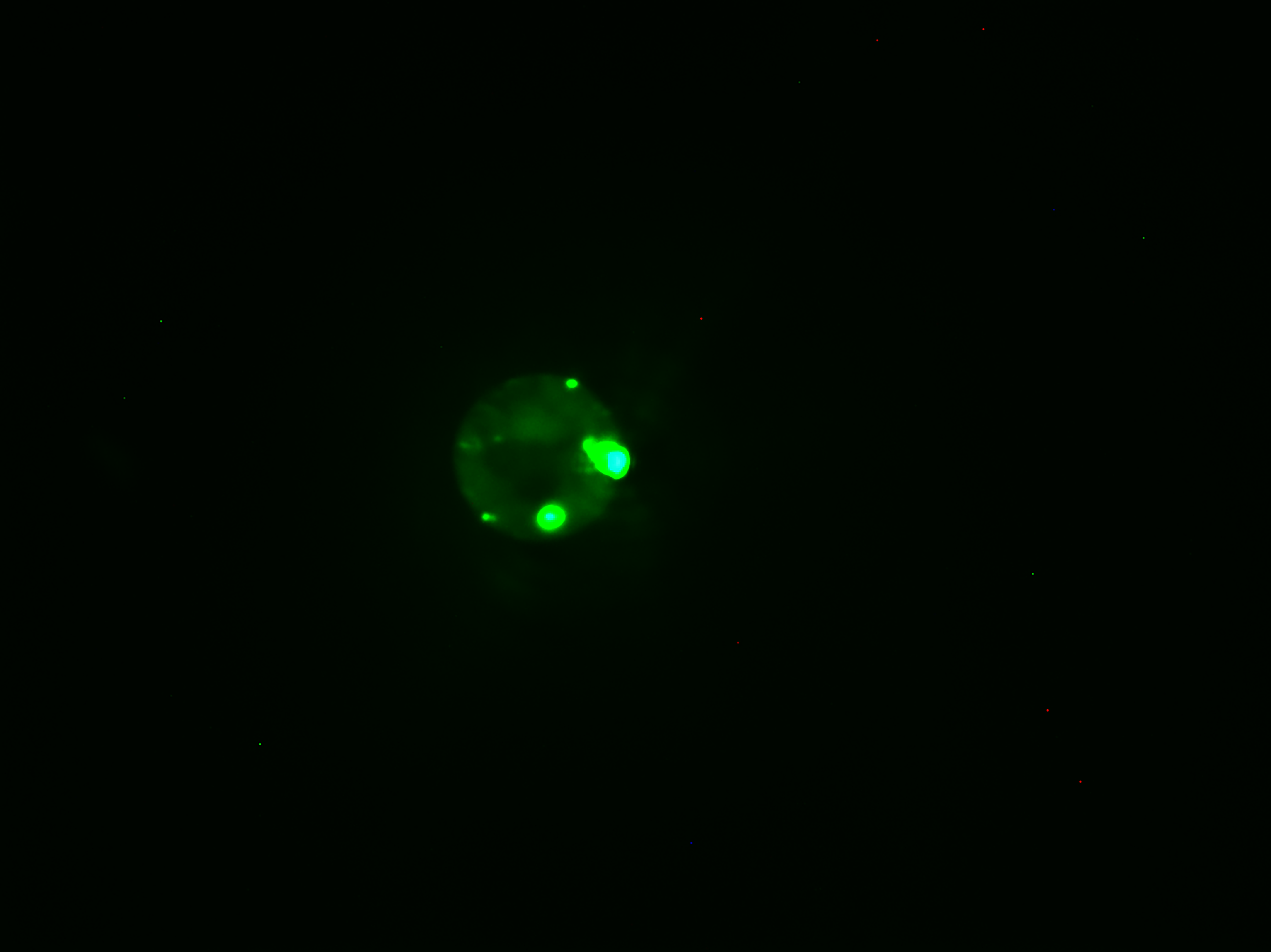

Supplement: Supplementary Data Sheet 1 — Twenty-nine differentially abundant metabolites between CK and transgenic Populus. [file DataSheet_1.zip › Data Sheet 1/Raw Data1093656/Fig.4/FLS/┼─╔π-1632.tif]

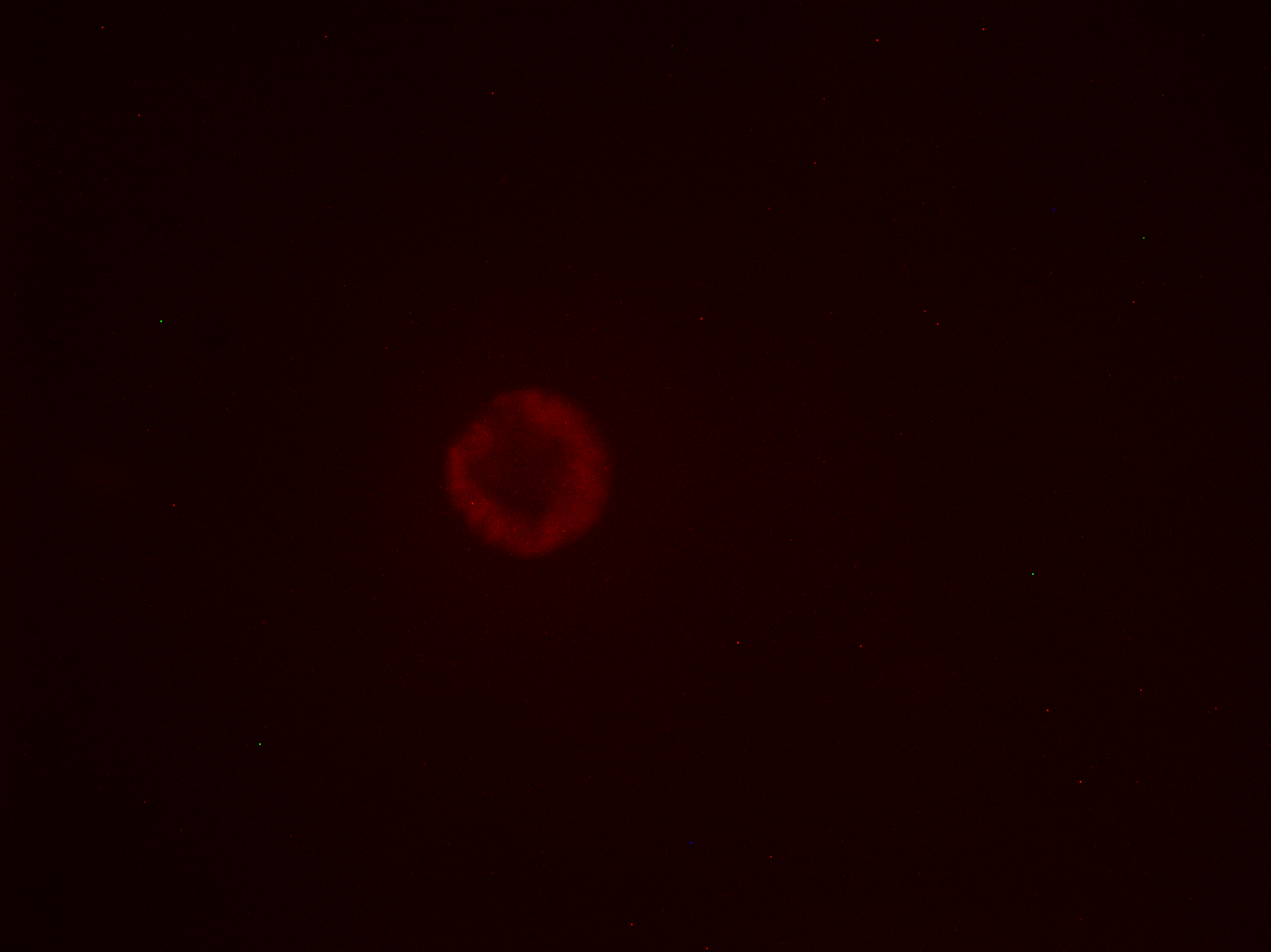

Supplement: Supplementary Data Sheet 1 — Twenty-nine differentially abundant metabolites between CK and transgenic Populus. [file DataSheet_1.zip › Data Sheet 1/Raw Data1093656/Fig.4/FLS/┼─╔π-1633.tif]
