# Supplementary material for: GbFLSa overexpression negatively regulates proanthocyanin biosynthesis
Source: Front Plant Sci. 2023 Feb 15;14:1093656. doi: 10.3389/fpls.2023.1093656 (PMC9975577; doi:10.3389/fpls.2023.1093656)
Supplement: Supplementary file 2 [file Table_1.docx]

**Supplemental Table S1**: Primers for *GbFLSa* cloning, RT-qPCR and vector construction

| **Primer_ID** | **Forward PCR primer (5**'**-3**'**)** | **Reverse PCR primer (5**'**-3**'**)** |
| --- | --- | --- |
| *GbFLSa*_5'OUTER | CTAATACGACTCACTATAGGGCAAGCAGTGGTATCAACGCAGAGT | TTCCCCTTGTGGTTGTTTTCTTC |
| *GbFLSa*_5'INNER | CTAATACGACTCACTATAGGGC | AAATCTCATACCTACACCATAC |
| *GbFLSa*_3'OUTER | GGAGTTCAAGCGGTTAGCAAAAG | ACTCTGCGTTGATACCACTGCTTGCCCTATAGTGAGTCGTATTAG |
| *GbFLSa*_3'INNER | GTTGGTCATCTCTCCTCTTGT | GCCCTATAGTGAGTCGTATTAG |
| *GbFLSa*_ORF | ATGGGTTCGAAACACATGG | CCCTTTATACTGTGTCTTAG |
| *GbFLSa*_qPCR | TTTGGACTGGCACCTCATTCT | TCTTGTACTTGCCGTTGCTC |
| BP detection | ATGAAGAGCTTTGCTAGT | TAATACGACTCACTATAGGG |
| LR detection | CGCACAATCCCACTATCCTT | CTAGGATAGCTTCCAAGCTTTACCA |
| HA | GCGCCCACAAGTTTGTACA | TTAAAGACTAGCATAATCTGGAAC |
| DFR1 | CCTGACAGCACTTTCATTGA | ACACGCCAAATTCTCATCAA |
| DFR2 | GGATTTTATCAGCGTCATACCACC | AGACAACACTCGCCAAATCCTC |
| DFR3 | GTGCYGTGGARACATGCAGAGAAA | GCCTTGCACTACAAGCATGGTACA |
| LAR1 | CAATCAATGGCCYTGATGAT | TGTCGTCCAAGAAAAAGAGA |
| LAR2 | TACTGACAGGGAAAACACGGAG | GTCGTAGTAAGGCCAAGAAGCA |
| LAR3 | CCTCCAAGCAATCTGCTAAGCACT | TCATGGTGCCATTCTTGTTCGCTG |
| ANS1 | GGTGACACTRTTGAGATCTT | CCATTTCAACGACATASCTT |
| ANS2 | ACTACTACCCCAAGTGCCCTCA | ACAATCTCTGCCAATGGCTTG |
| ANS3 | CGATTTTGTGTTCGCAAAAGAT | GAAAGCAAGTGCATCGGAAT |
| Reference gene of *Ginkgo* | GGTGCCAAAAAGGTGGTCAT | CAACAACGAACATGGGAGCAT |
| Reference gene of *Populus* | GGCAAGGAGAAGGTACACAT | CAATCACACGCTTGTCAATA |
